# Assessing Change in Stone Burden on Baseline and Follow-Up CT: Radiologist and Radiomics Evaluations

**DOI:** 10.3390/jimaging12010013

**Published:** 2025-12-27

**Authors:** Parisa Kaviani, Matthias F. Froelich, Bernardo Bizzo, Andrew Primak, Giridhar Dasegowda, Emiliano Garza-Frias, Lina Karout, Anushree Burade, Seyedehelaheh Hosseini, Javier Eduardo Contreras Yametti, Keith Dreyer, Sanjay Saini, Mannudeep Kalra

**Affiliations:** 1 Department of Radiology, Massachusetts General Hospital, Boston, MA 02114, USA; pkaviani@mgh.harvard.edu (P.K.);; 2 MGB Center for Clinical Data Science, Boston, MA 02120, USA; 3 Department of Radiology and Nuclear Medicine, University Medical Center Mannheim, Medical Faculty Mannheim, Heidelberg University, 68167 Mannheim, Germany; 4 Siemens Medical Solutions USA Inc., Malvern, PA 19355, USA

**Keywords:** kidney stone, renal calculi, computed tomography, kidney stone burden

## Abstract

This retrospective diagnostic accuracy study compared radiologist-based qualitative assessments and radiomics-based analyses with an automated artificial intelligence (AI)–based volumetric approach for evaluating changes in kidney stone burden on follow-up CT examinations. With institutional review board approval, 157 patients (mean age, 61 ± 13 years; 99 men, 58 women) who underwent baseline and follow-up non-contrast abdomen–pelvis CT for kidney stone evaluation were included. The index test was an automated AI-based whole-kidney and stone segmentation radiomics prototype (Frontier, Siemens Healthineers), which segmented both kidneys and isolated stone volumes using a fixed threshold of 130 Hounsfield units, providing stone volume and maximum diameter per kidney. The reference standard was a threshold-defined volumetric assessment of stone burden change between baseline and follow-up CTs. The radiologist’s performance was assessed using (1) interpretations from clinical radiology reports and (2) an independent radiologist’s assessment of stone burden change (stable, increased, or decreased). Diagnostic accuracy was evaluated using multivariable logistic regression and receiver operating characteristic (ROC) analysis. Automated volumetric assessment identified stable (*n* = 44), increased (*n* = 109), and decreased (*n* = 108) stone burden across the evaluated kidneys. Qualitative assessments from radiology reports demonstrated weak diagnostic performance (AUC range, 0.55–0.62), similar to the independent radiologist (AUC range, 0.41–0.72) for differentiating changes in stone burden. A model incorporating higher-order radiomics features achieved an AUC of 0.71 for distinguishing increased versus decreased stone burdens compared with the baseline CT (*p* < 0.001), but did not outperform threshold-based volumetric assessment. The automated threshold-based volumetric quantification of kidney stone burdens provides higher diagnostic accuracy than qualitative radiologist assessments and radiomics-based analyses for identifying a stable, increased, or decreased stone burden on follow-up CT examinations.

## 1. Introduction

Computed tomography (CT) is the imaging modality of choice for assessing patients with suspected kidney stones and for assessing stone burden over time. Given the high prevalence of kidney stones in the US population and up to a 50% recurrence rate following treatment or spontaneous passage, their imaging evaluation plays an important role in treatment planning [1]. As per the National Health and Nutrition Examination Survey (NHANES), up to 6.3% of men and 4.1% of women had kidney stones in the United States [2]. Although prevalence varies from 1–5% in Asia, 5–9% in Europe, and 7–13% in North America, kidney stones represent a global health issue [3].

While detection of kidney stones beyond differentiation from vascular calcifications is not a difficult task, the comparison of stone burdens over serial imaging, particularly in the settings of complex or multiple kidney stones, is subjective, tedious, and time-consuming. Overall, kidney stone burden affects the choice of treatment despite discrepancies in clinical indications and efficacy across different treatment options [4]. The shockwave lithotripsy (SWL) procedure is the best treatment option for larger kidney stones (particularly for stones > 1 cm). Treatment of kidney stones between 1–2 cm in size is controversial, although percutaneous nephrolithotomy (PCNL) is generally regarded as the preferred treatment for stones smaller than 2 cm [5]. The invasive nature, high cost, and risk of complications with surgical and interventional procedures emphasize the need for accurate stone size estimation.

Low-dose, non-contrast abdomen–pelvis CT is the preferred method for evaluating kidney stones and their burden [6,7]. Prior studies have evaluated radiomics and artificial intelligence-based systems for kidney stone volumes [8]. However, most radiomics platforms are complex and not cleared by the US Food and Drug Administration (FDA). On the other hand, most AI tools either lack proof of generalizability or multicenter evaluation for reliable clinical use [9]. Most costs related to the use of AI tools in imaging are also not reimbursable.

There are FDA-cleared tools for quantifying and/or tracking the longitudinal burden of imaging abnormalities, such as lung nodules, emphysema, and coronary calcification, but to the best of our knowledge, there are no AI tools to track changes in stone burden over serial imaging. The latter helps assess treatment effectiveness and choice and is therefore an important indication for follow-up scanning in patients with known kidney stones. While reporting, radiologists primarily document changes in stone burden using the greatest stone dimensions [10]. However, prior studies report substantial variations between urologic and radiologic CT interpretations of kidney stone burdens, which can influence decisions regarding the best treatment approach [11]. Therefore, we compared radiologists’ qualitative assessments and radiomics-based analyses of kidney stone burdens with an automated, threshold-based volumetric measurement derived from AI-assisted whole-kidney and stone segmentation on CT, with particular emphasis on evaluating interval changes in stone burden.

## 2. Materials and Methods

### 2.1. Ethical Approval and Disclosures

We received approval from the Human Research Committee of the institutional review board at Massachusetts General Brigham (protocol number: 2020P003950, approval date: 23 December 2020) for a retrospective study of patients with kidney stones on the baseline and follow-up abdomen–pelvis CT examinations. The need for written informed consent was waived. This study was compliant with the Health Insurance Portability and Accountability Act (HIPAA). A co-author received a research grant from Siemens Healthineers.

### 2.2. Patients

To identify eligible patients, we used our proprietary radiology report search engine, rendering a query with the following keywords: “adult patients”, “non-contrast abdomen–pelvis CT with kidney stone protocol”, and “kidney stones”. The search yielded 1270 CT examinations that mention kidney stones between July and December 2020.

A study coinvestigator reviewed all radiology reports to exclude CT examinations with renal stents (*n* = 58), in situ pigtail catheters (*n* = 43), nephrostomy tubes (*n* = 76), surgical clips (*n* = 48), and metal-related streak artifacts projecting over the kidneys (*n*= 23). In addition, patients (*n* = 579) with kidney stones < 5 mm were excluded. The final sample size was 157 adult patients (mean age 61 ± 13 years; 99 males and 58 females), each with two non-contrast, abdomen–pelvis CTs (314 CT examinations).

### 2.3. CT Protocols

All abdomen–pelvis CT examinations were performed on one of the four multidetector-row CT scanners using the kidney stone protocol: 175 examinations on a 64-section CT scanner (GE Discovery 750 HD; GE Healthcare, Waukesha, WI, USA; baseline CT = 92, follow-up CT = 83), 37 examinations on a 128-section scanner (Philips iCT; Philips Healthcare, Cleveland, OH, USA; baseline CT = 15, follow-up CT = 22), 101 examinations on a 256-section scanner (Siemens Definition Flash; Siemens Healthineers, Forchheim, Germany; baseline CT = 49, follow-up CT = 52), and one exam on a 320-section scanner (Canon Aquilon One; Canon Medical Systems, Otawara, Tochigi, Japan; single baseline CT).

All CT examinations were performed using a single-energy technique in helical scan mode, 100–120 kV, automatic exposure control, 0.9–1.375:1 pitch, 0.5–0.8 s gantry rotation time, and 5 mm section thickness with 5 mm section intervals. DICOM images of each CT were de-identified and exported offline for processing on an offline Radiomics prototype (eXamine, Siemens Healthineers, Erlangen, Germany).

### 2.4. Stone Measurement

DICOM image datasets of each patient were organized into two paired subfolders, one with baseline CT images and the other with follow-up CT images. Each dataset was separately processed with the Radiomics prototype using an identical method. First, we uploaded individual CT exams into the prototype and clicked the Kidney Isolation icon. The prototype used an AI-based organ identification and segmentation capability to segment the right and left kidneys, including the pelvicalyceal collecting system. For segmenting the volume of interest (VOI) confined to the stones, we applied a threshold of 130 HU within the segmented kidneys. Because the volumetric measurements were derived from a fixed threshold-based method, the analysis is independent of proprietary radiomics models and reproducible with alternative segmentation tools. The proprietary software was thus used only for segmentation, rather than for determining the study’s core quantitative results. The stone VOI included all kidney stones and their entire volume. A study coinvestigator (PK, who has four years of post-doctoral research experience in radiology) reviewed all renal and kidney stone segmentations. Cases that required the manual editing of renal volumes or kidney stones were recorded.

**Figure 1:**
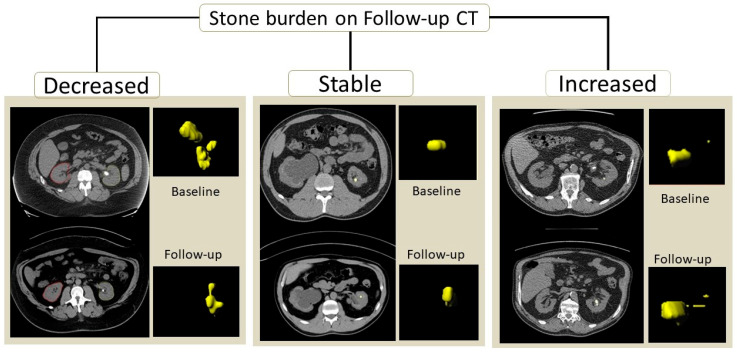
Transverse CT and “stone segmentation” volume-rendered images of three patients with decreased, stable, and increased stone burdens on a follow-up CT compared to the baseline CT.

After confirming and/or editing the stone VOIs, we extracted the radiomics features over the stone VOIs from the same prototype. We considered radiomics as the ground truth for kidney stone volumes. In the 157 patients, radiomics was estimated separately for each of the 314 kidneys on baseline and follow-up CT exams. Kidney stone burden was classified as decreased on follow-up CT in 108 kidneys, increased in 109 kidneys, and stable in the remaining 44 kidneys. Figure 1 illustrates the kidney stone segmentation in patients with an increased, decreased, or stable calculi burden.

The decision regarding stone burden change or stability was based on segmented volumes of stones obtained separately for each kidney on baseline and follow-up CT examinations. A change in stone volume of less than 15% was regarded as evidence of a stable stone burden, whereas a greater than 15% increase or decrease in stone volume upon follow-up CT was deemed to indicate an increased or decreased stone burden [12].

### 2.5. Qualitative Evaluation

We extracted information on the reporting radiologists’ description of stability, increase, or decrease in stone burden from the radiology reports. Separately, a radiologist (MKK, who has 29 years of experience in CT) commented on the stability, increase, or decrease in stone burden from a side-by-side comparison of baseline and follow-up CT examinations. The radiologists were not aware of the radiomics-based classification of stone burden. We also recorded details pertaining to any interventional procedures performed between the two CT examinations from the electronic medical records.

### 2.6. Radiomics Feature Selection

Radiomics features were extracted using the commercial research prototype. The software automatically generates standardized first-order, texture, and higher-order features from segmented stone volumes, with feature definitions, extraction parameters, and feature combinations predefined by the vendor and not user-modifiable.

As a result, feature selection and weighting reflected the internal design of the prototype rather than investigator-driven optimization. Accordingly, the radiomics component of this study was designed to provide an exploratory, hypothesis-generating evaluation of stone characteristics, rather than to develop or fine-tune a customized predictive model.

### 2.7. Statistical Analysis

Data were analyzed with R Statistical Software version 4.5.2 (R Statistical Computing, http://www.R-project.org, R Foundation for Statistical Computing, Vienna, Austria, accessed on 10 June 2021). Since intervention or spontaneous stone removal can change the stone volume in a single kidney, data analysis was performed on a per-kidney basis for assessing stone burden changes. Multiple logistic regression analysis was used to assess changes in the burden of kidney stones. We set the area under the curve (AUC) as the output for the precision–recall curve (PRC) analysis. PRC was preferred over receiver operating characteristic (ROC) analysis due to the asymmetric distribution of stone burden in the stable and change categories.

For summary statistics, the tableone package (version 0.13.2) was utilized. For the comparison of means, a Student’s t-test was performed. For the comparison of categorial variables, the chi-squared test was performed. For the visualization of data in boxplots, ggplot2 (version 3.4.1.) was used. Furthermore, the visualization of all radiomics features in a heatmap was performed with the ComplexHeatmap package (version 2.14.0), utilizing hierarchical clustering. The normalization of data was performed with z-score normalization.

This study was conducted and reported in accordance with the Standards for Reporting of Diagnostic Accuracy Studies (STARD) guidelines, and the completed STARD checklist is provided in the Supplemental Materials.

## 3. Results

### 3.1. Stone Measurement and Segmentation

Overall, 475 stone measurements, 248 at baseline and 227 at follow-up, were included in the analysis. For baseline and follow-up, there was no significant difference in sex, age, stone volume, max diameter, slice, thickness, tube voltage, tube current, or scanner manufacturer (all *p* > 0.05). However, there was a difference in the scanner model (*p* = 0.045). A per-stone overview is shown in Table 1. An overview table for all radiomics features is shown in the Supplemental Materials. On a patient-based assessment, in the 157 patients, the stone burden was stable in 15 patients upon follow-up CT, decreased in 58 patients, and increased in 55 patients. In the remaining 29 patients, the stone burden increased in one kidney and decreased in the other kidney. Of the 314 kidneys, 44 had a stable stone burden, 109 had an increased stone burden, and 108 had a decreased stone burden upon follow-up CT examinations (with a mean interval of 19 ± 28 months between baseline and follow-up CTs). In 53 patients, kidney stones were unilateral on the baseline and/or follow-up CT examinations.

Among the 53 patients with a decreased stone burden on their follow-up CT, 13 patients had an interval treatment procedure (including laser lithotripsy and extracorporeal shockwave therapy) between their baseline and follow-up CTs. Among the fifty-five patients with an increased stone burden upon follow-up imaging, only seven patients (7/55) had an intervention procedure (laser lithotripsy or ureteral stent placement). Patients with a stable stone burden had no interventional treatment for kidney stones between their two CT examinations.

The threshold-based segmentation of kidney stone volume was successful in isolating all kidney stones and did not require any manual editing. The manual adjustment of automatic kidney segmentation was needed in about 15% of CT examinations (47/314 baseline and follow-up CTs). This involved editing the renal contours to include the entire renal parenchyma and exclude the extra-renal adjacent soft tissues. Stone volumes and maximum dimensions are summarized in Table 2.

### 3.2. Radiologists’ Assessment of Stone Burden

Compared to quantitative stone volumes, the clinical interpretation of stone burden change and stability on follow-up CT radiology reports had low AUCs (stable stone burden, AUC 0.55; decreased stone burden, AUC 0.59; increased stone burden, AUC 0.62). The corresponding AUCs for the independent radiologist were not substantially different (stable stone burden, AUC 0.41; decreased stone burden, AUC 0.61; increased stone burden, AUC 0.72). There was high agreement between the radiology reports and the independent radiologist (unweighted kappa = 0.96, Table 3).

**Figure 2:**
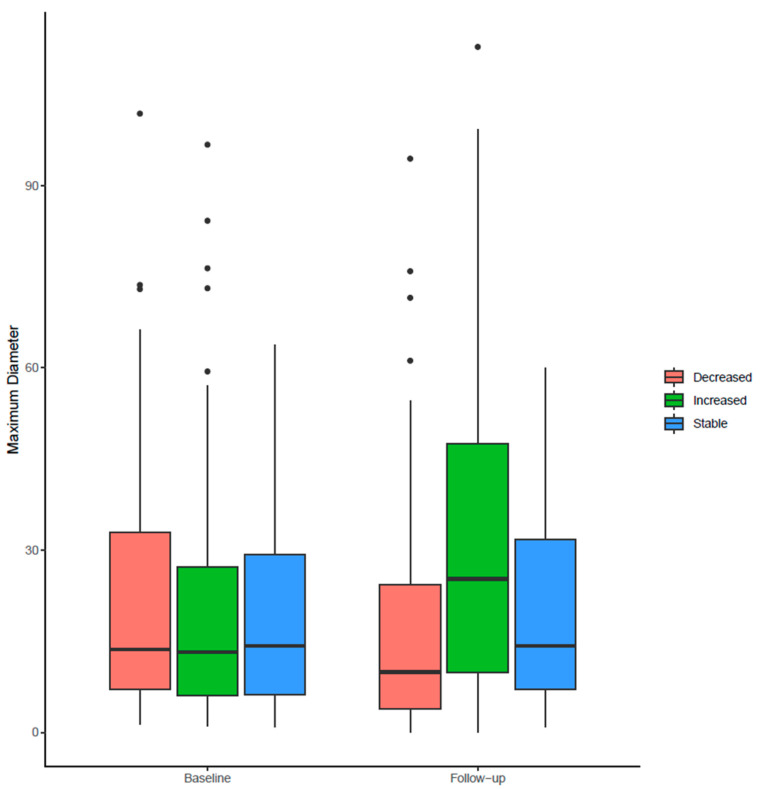
Boxplot of decreased, increased, and stable groups, comparing baseline with follow-up. Each dot represents an individual stone measurement at a given time point (baseline or follow-up), and paired dots connected by lines correspond to the same stone across examinations.

The maximum diameter in association with the independent radiologist’s readings is visualized in Figure 2.

**Figure 3:**
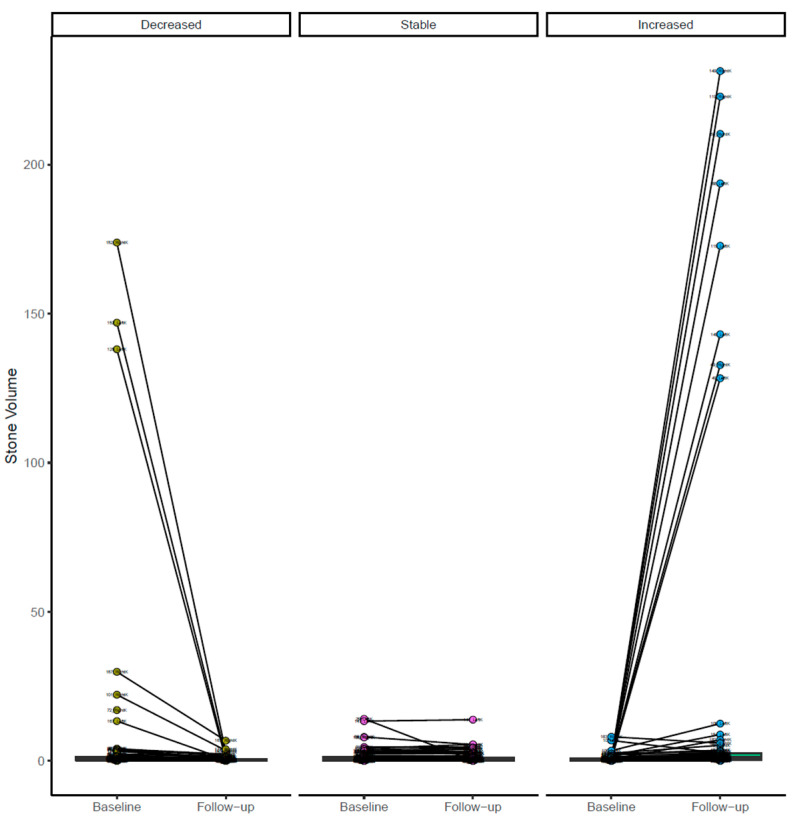
Illustration of the changes in the decreased, stable, and increased groups when comparing the abdomen–pelvis CT exam to the baseline exam.

The change in stone volume dependent on the radiologist’s reading is shown in Figure 3, showing high agreement.

### 3.3. Radiomics

**Figure 4:**
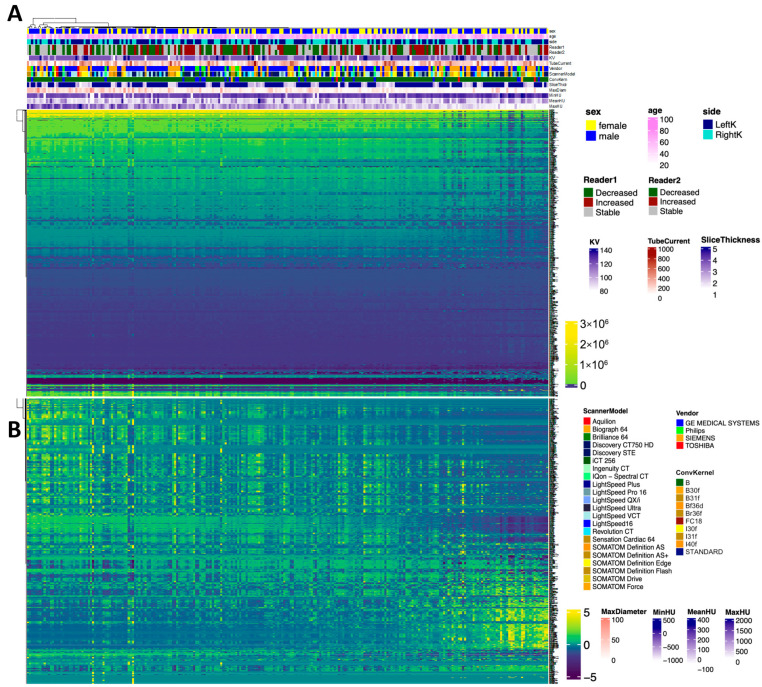
Heatmaps of non-normalized (**A**) and normalized (**B**) data illustrating radiomics features across the following variables: sex, age, kidney side, reader 1, reader 2, scanner parameters (KV, tube current, slice thickness, vendors, and scanner models), stone max diameter, and the minimum, mean, and maximum Hounsfield unit.

The association of baseline radiomics features with multiple clinical and imaging parameters is visualized as a heatmap in Figure 4 for both non-normalized (4A) and z-score-normalized (4B) radiomics features.

**Figure 5:**
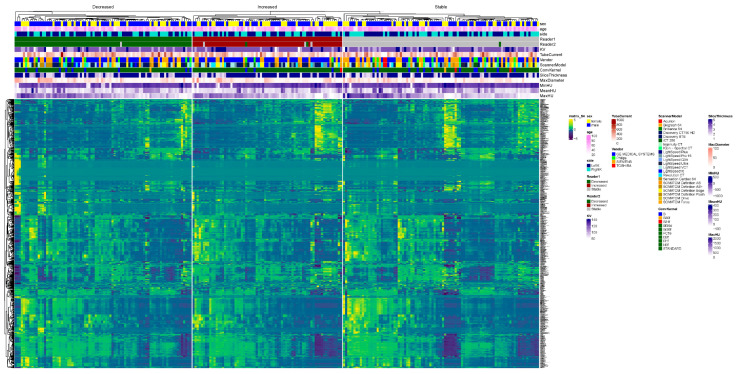
Heatmap summarizing the radiomics features across the decreased, increased, and stable groups, comparing follow-up abdomen–pelvis exams to baseline exams, as well as the following parameters: sex, age, kidney side, reader 1, reader 2, scanner parameters (KV, tube current, slice thickness, vendors, and scanner models), stone max diameter, and the minimum, mean, and maximum Hounsfield unit.

Hierarchical clustering of stones was performed for both examples based on the non-normalized radiomics values. There was no significant difference in the radiomics of kidney stones with a stable burden on baseline and follow-up CT examinations (*p* > 0.1). Baseline radiomics features were then split according to the qualitative reads of the independent radiologist (Figure 5), which revealed a high degree of variability within the groups. However, there were significant statistical differences in higher-order statistics between patients with increased or decreased stone burdens on follow-up CTs as compared to their respective baseline CT examinations (*p* < 0.001).

Although the best selected radiomics feature combinations provided by the prototype showed statistically significant associations with changes in stone burden (Table 4) (*p*< 0.001), their overall discriminative performance remained modest in this cohort (AUC ≤ 0.71).

**Figure 6:**
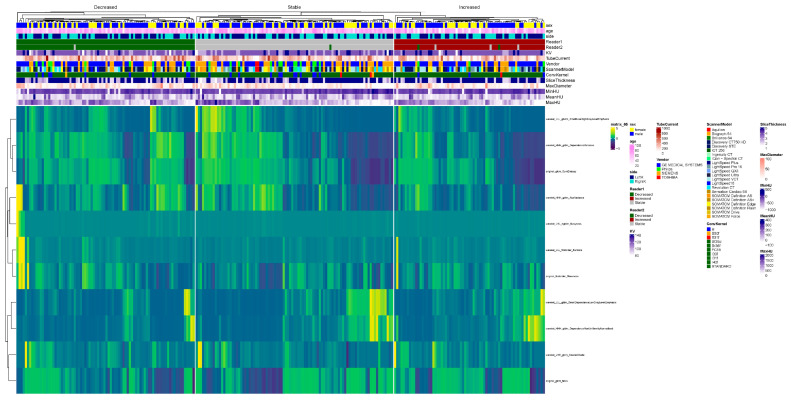
Heatmap illustrating the ten best radiomics features in the decreased, stable, and increased groups, comparing follow-up abdomen–pelvis CT examinations to baseline examinations across the following parameters: sex, age, kidney side, reader 1, reader 2, scanner parameters (KV, tube current, slice thickness, vendors, and scanner models), stone max diameter, and the minimum, mean, and maximum Hounsfield unit.

Selected baseline radiomics features associated with increased and/or decreased stone burdens during follow-up were also visualized in a heatmap (Figure 6).

## 4. Discussion

Our study demonstrates that AI-based kidney segmentation and HU-based stone volume determination can help assess stability and changes in stone burden on follow-up CT examinations. In contrast to qualitative interpretation by a radiologist, which showed limited reliability in differentiating stable, increased, or decreased stone burdens, this automated approach allows for a more objective assessment over time. Importantly, about 85% of abdomen–pelvis CTs did not require any manual editing of the segmented kidneys or kidney stones, which implies that in most cases, it is possible to automate stone burden assessments without the need for modifying segmentations.

We found tremendous inter-radiologist variation in the evaluation of stone burden over serial CT exams. The agreement on kidney stone burden between two radiologists was only 50% in our study due to the presence of substantial discrepancies in categories with stable, increased, and decreased stone burdens. Other studies have also reported substantial inter-observer variations and disagreements [13,14,15,16], although we found a much higher discrepancy rate than the previously reported 2–29% discrepancy rates. Compared with Abujudeh et al.’s [17] reported 26–32% inter-radiologist discrepancy for the detection and documentation of abdomen–pelvis CT findings, our study points out that qualitative stone burden assessments are prone to greater variation from the ground truth. Such variations likely stem from subtle changes in stone morphology or orientation, which are difficult to assess visually, as opposed to a threshold-based method for isolating and quantifying stone volumes. Reading quality and diagnostic interpretation accuracy are crucial when it comes to determining an appropriate patient treatment pathway.

Tzou et al. have also reported considerable variation between radiologists’ and urologists’ interpretations of stone burdens on CTs [18]. As noted in our study, even though there are best-practice radiological criteria for measuring and reporting stone burdens, the amount of detail in the radiological report varies, with only 7% of reports describing a change in stone volume in the three-dimensional planes [19]. Thus, the major clinical implication of our study is the need to either improve the metrics radiologists use for describing their opinion on stone burden evolution on follow-up imaging or adopt a more quantitative method for documenting kidney stone burdens, such as the one reported in our study. At the time of writing this manuscript, the method of estimating the kidney stone burden with our prototype does not have US FDA clearance. In addition, radiomics features have been described as partly unstable with respect to scanner hardware, scanning protocols, and reconstruction algorithms (10.1002/mp.13808). In this study, there was a high degree of feature variability associated with the mean and maximum Hounsfield intensity. These explain the poor radiomics performance in our study. Unstable radiomics may be addressed using novel CT imaging techniques, such as photon-counting detector CTs (https://www.nature.com/articles/s41598-022-22877-8 (accessed on 1 August 2025)) or deep learning-based reconstruction algorithms (https://link.springer.com/article/10.1007/s00330-022-08592-y (accessed on 1 August 2025)).

Our study also highlights the limitations of both subjective radiologists and radiomics-based approaches when monitoring longitudinal stability versus changes in the stone burden. While subjective variations in longitudinal quantitative tasks are expected, radiomics likely suffer from variations in scanner types, acquisition, and reconstruction parameters between baseline and follow-up CT exams. Thus, we believe that a simple approach of first segmenting the kidneys and then applying a threshold to segment and quantify longitudinal stone burden should be the preferred approach in the absence of a robust AI technique that parses the renovascular calcification and other non-stone parenchymal calcification from renal calculi.

An additional limitation of this study is the absence of a formal analysis examining the radiologist’s accuracy relative to the magnitude of the stone burden change. Radiologists’ interpretations were limited to categorical assessments of stability, increase, or decrease, without stratification by the extent of volumetric change. It is likely that larger changes in the stone burden are more readily recognized, whereas smaller changes may fall below the threshold of reliable visual detection. Evaluating performance across the strata of volumetric change would necessitate a substantially larger cohort and a study design specifically powered for such subgroup analyses. This represents an important area for future investigation.

Although a proprietary research prototype was used for kidney segmentation and radiomics feature extraction, the primary quantitative analysis in this study was based on threshold-defined volumetric stone burden measurements. This analysis relies on fixed Hounsfield unit thresholds and voxel-based volume calculations rather than learned feature representations or adaptive model outputs, making it fully deterministic and reproducible. Accordingly, the use of proprietary software does not affect the reproducibility of this study’s main quantitative findings, and comparable volumetric results could be obtained using alternative open-source segmentation tools implementing the same threshold-based approach. Nevertheless, we acknowledge that the use of proprietary AI software limits the direct inspection of internal model architecture and feature extraction processes, thereby constraining methodological transparency. As a potential mitigation strategy, several publicly available multi-organ segmentation tools for kidney and urinary bladder segmentation could be applied in future studies to independently replicate the segmentation workflow and validate the robustness of our inferences. Other software, such as StoneChecker (Imaging Biometrics, Wisconsin, USA), help to measure stone dimensions and volumes, as well as differentiate urate and non-urate stones. We did not find any other CE- or US FDA-cleared AI tools for kidney stone burden analysis.

Additionally, this study was limited to patients from the northeast region of the United States and may not be generalizable across other regions. Second, the distribution of CT examinations was asymmetric, with a limited number of patients with a stable stone burden on follow-up CT examinations. Likewise, examinations from two of the four CT vendors represented a minority of cases. Moreover, we used a threshold-based volume estimation from segmented stone VOIs as the standard of reference for testing the performance of the radiologists and radiomics. Although this is not an established standard of reference for stone burden quantification, coupled with visual verification of segmentation accuracy, we believe that it provided a robust and objective ground truth, especially in view of considerable variations in the description of stone burdens in radiology reports and by the independent radiologist. In addition, we did not formally assess the time or effort involved in the application of HU-based stone segmentation or radiomics analysis. Given the emphasis on reporting efficiency, such an assessment could help define its clinical implementation and use. However, prior publications suggest that organ segmentation and radiomics estimation can take as little as a few seconds [20]. Likewise, we did not assess the impact of section thickness or reconstruction technique and kernels on kidney segmentation and stone burden assessment, since most low-dose CT examinations for kidney stones at our site are reconstructed at a section thickness of 5 mm and soft kernel using commercial iterative reconstruction techniques. Prior studies report variations in radiomics with changes in reconstruction and acquisition parameters [21]. Additionally, we excluded kidney stones < 5 mm in our study. Although these stones are common, they were omitted because they are typically clinically insignificant and often pass spontaneously without intervention. Including them would have introduced substantial variability in detection and volumetric estimation, without meaningfully contributing to the assessment of clinically actionable stone burdens. Another limitation of this study relates to the use of a CT section thickness of 5 mm. While thinner reconstructions are increasingly used in current clinical practice and may mitigate partial-volume effects, our choice was dictated by the longitudinal retrospective nature of the cohort. During the study period, 5 mm reconstructions constituted the standard of care for the majority of baseline and follow-up examinations. Importantly, maintaining consistent reconstruction parameters across paired scans was necessary to ensure the reliable assessment of interval changes in stone burdens. It is possible that results obtained using thinner sections, particularly for radiomics-based analyses, may differ, and this remains an area for future investigation. Finally, the absence of a formal analysis examining the radiologist’s accuracy relative to the magnitude of stone burden change is another limitation. Radiologist interpretations were limited to categorical assessments of stability, increase, or decrease, without stratification by the extent of volumetric change. It is likely that larger changes in stone burden are more readily recognized, whereas smaller changes may fall below the threshold of reliable visual detection. Evaluating performance across the strata of volumetric change would necessitate a substantially larger cohort and a study design specifically powered for such subgroup analyses. This represents an important area for future investigation.

Compared to prior publications based on a single CT per patient stone burden, our study highlights the challenges associated with the longitudinal assessment of stone burdens.

In conclusion, the volumetric assessment of kidney stone burdens using fixed Hounsfield unit criteria provides a more robust and interpretable approach for differentiating stable, increased, or decreased stone burdens on follow-up CTs compared with qualitative radiologist interpretations and exploratory radiomics-based analyses.

## Figures and Tables

**Table 1:** Overview of clinical and scanner-related parameters.

	Overall	Baseline	Follow-Up	*p*-Value
	Frequency (Percentage%)	Frequency (Percentage%)	Frequency (Percentage%)	
Patients	475	248	227	
Sex = male (%)	294 (61.9)	153 (61.7)	141 (62.1)	1.000
Age (mean (SD))	61.18 (13.21)	61.21 (13.24)	61.15 (13.20)	0.958
Object volume (mean (SD))	5.11 (26.45)	3.09 (16.99)	7.32 (33.80)	0.082
Max diameter (xz axis) (mean (SD))	27.92 (27.37)	28.24 (27.77)	27.57 (26.98)	0.792
Max diameter (yz axis) (mean (SD))	33.12 (32.36)	31.44 (32.25)	34.95 (32.44)	0.237
Number of slices (mean (SD))	134.15 (70.74)	131.04 (62.28)	137.55 (78.94)	0.317
Voxel spacing Z (mean (SD))	4.03 (1.34)	4.04 (1.32)	4.02 (1.37)	0.915
Slice thickness (mean (SD))	4.03 (1.34)	4.04 (1.32)	4.03 (1.36)	0.950
KVP (mean (SD))	116.76 (11.87)	117.26 (12.91)	116.21 (10.63)	0.338
X-ray tube current (mean (SD))	278.83 (152.13)	277.63 (147.52)	280.15 (157.34)	0.857
Manufacturer (%)				0.241
GE	262 (55.2)	143 (57.7)	119 (52.4)	
Philips	55 (11.6)	24 (9.7)	31 (13.7)	
Siemens	156 (32.8)	79 (31.9)	77 (33.9)	
Canon	2 (0.4)	2 (0.8)	0 (0.0)	
Manufacturer model name (%)				0.045
Aquilion	2 (0.4)	2 (0.8)	0 (0.0)	
Biograph 64	11 (2.3)	3 (1.2)	8 (3.5)	
Brilliance 64	2 (0.4)	2 (0.8)	0 (0.0)	
Discovery CT750 HD	95 (20.0)	48 (19.4)	47 (20.7)	
Discovery STE	10 (2.1)	8 (3.2)	2 (0.9)	
iCT 256	21 (4.4)	9 (3.6)	12 (5.3)	
Ingenuity CT	1 (0.2)	1 (0.4)	0 (0.0)	
IQon-Spectral CT	31 (6.5)	12 (4.8)	19 (8.4)	
LightSpeed Plus	3 (0.6)	3 (1.2)	0 (0.0)	
LightSpeed Pro 16	25 (5.3)	12 (4.8)	13 (5.7)	
LightSpeed QX/i	1 (0.2)	1 (0.4)	0 (0.0)	
LightSpeed Ultra	6 (1.3)	3 (1.2)	3 (1.3)	
LightSpeed VCT	70 (14.7)	38 (15.3)	32 (14.1)	
LightSpeed16	15 (3.2)	10 (4.0)	5 (2.2)	
Revolution CT	36 (7.6)	20 (8.1)	16 (7.0)	
Revolution Frontier	1 (0.2)	0 (0.0)	1 (0.4)	
Sensation Cardiac 64	2 (0.4)	2 (0.8)	0 (0.0)	
SOMATOM Definition AS	8 (1.7)	5 (2.0)	3 (1.3)	
SOMATOM Definition AS+	3 (0.6)	2 (0.8)	1 (0.4)	
SOMATOM Definition Edge	53 (11.2)	29 (11.7)	24 (10.6)	
SOMATOM Definition Flash	15 (3.2)	13 (5.2)	2 (0.9)	
SOMATOM Drive	4 (0.8)	2 (0.8)	2 (0.9)	
SOMATOM Force	60 (12.6)	23 (9.3)	37 (16.3)	

**Table 2:** Volumes (ml) and maximum dimension (mm) of kidney stones on baseline and follow-up CTs in patients with stable, increased, or decreased stone burdens. There was no significant difference in volumes and the maximum diameter of stones in patients with a stable burden on follow-up CTs (*p* value > 0.1). In contrast, both the decreased and increased stone burden groups demonstrated a significant change in stone volumes and diameters (*p* value < 0.001).

	Baseline CT	Follow-Up CT
	Maximum Diameter (mm)	Maximum Diameter (mm)
Stable group	24.43 ± 21.01	24.72 ± 20.76
Decreased group	29.16 ± 25.91	22.78 ± 23.79
Increased group	27.33 ± 28.51	38.56 ± 28.56
	Volume (mL)	Volume (mL)
Stable group	1.24 ± 2.39	1.3 ± 1.87
Decreased group	7.22 ± 28.91	0.61 ± 1.12
Increased group	0.65 ± 1.05	23.07 ± 60.09

**Table 3:** Summary of the inter-reader agreements over decreased, increased, and stable groups and Cohen Kappa and weighted Kappa correlation coefficients (with confidence boundaries).

Agreement Between Radiologists	Decreased	Increased	Stable
Decreased	148	0	2
Increased	4	132	6
Stable	2	0	181
	Kappa (Confidence Boundaries)		
	Lower	Estimate	Upper
Unweighted Kappa	0.93	0.96	0.98
Weighted Kappa	0.94	0.96	0.99

**Table 4:** Best combination of radiomics for assessing patients with decreased or increased stone burdens on follow-up CT as compared to their baseline CT exams. All areas under the curve (AUCs) were statistically significant (*p*< 0.001). In contrast, there were no differentiating radiomics in patients with stable stone burdens.

Stone Burden	Best Combination of Radiomics	AUC
Decreased stone burden	Wavelet-HHH-GLDM-Dependence Variance + wavelet-HLL-GLDM-Small Dependence Emphasis + wavelet-LLH-NGTDM-Complexity	0.69
	Wavelet-HHH-GLDM-Dependence Variance + wavelet-HLL-GLDM-Small Dependence Emphasis	0.68
Increased stone burden	Wavelet-LHH-first order-Total Energy + wavelet-LHL-GLDM-Dependence Variance + wavelet-LHL-GLDM-Dependence Variance	0.71
	Wavelet-LHH-first order-Total Energy + wavelet-LHL-GLDM-Dependence Variance	0.65

## Data Availability

The original contributions presented in this study are included in the article and Supplementary Materials. Further inquiries can be directed to the corresponding author.

## Supplementary Materials

The following supporting information can be downloaded at: https://www.mdpi.com/article/10.3390/jimaging12010013/s1, File S1: STARD checklist summarizing compliance with reporting standards for diagnostic accuracy studies and indicating the manuscript pages where each item is reported. File S2: Comparison of radiomics features between baseline and follow-up CT examinations. Reported p values indicate the statistical significance of differences observed between time points.
